# Low-frequency electromagnetic fields combined with tissue engineering techniques accelerate intervertebral fusion

**DOI:** 10.1186/s13287-021-02207-x

**Published:** 2021-02-17

**Authors:** Weigang Li, Chunwei Huang, Tian Ma, Jiachen Wang, Wenbin Liu, Jiyuan Yan, Gaohong Sheng, Ruizhuo Zhang, Hua Wu, Chaoxu Liu

**Affiliations:** 1 Department of Orthopedics, Tongji Hospital, Tongji Medical College, Huazhong University of Science and Technology, Wuhan, 430030 Hubei China; 2 Department of Thyroid and Breast Surgery, The Central Hospital of Wuhan, Tongji Medical College, Huazhong University of Science and Technology, Wuhan, 430030 Hubei China; 3 Department of Hematology, Tongji Hospital, Tongji Medical College, Huazhong University of Science and Technology, Wuhan, 430030 Hubei China; 4 Department of Orthopedics, Xiangya Hospital of Central South University, Changsha, 410008 Hunan China

**Keywords:** Sinusoidal electromagnetic field, Lumbar degenerative disease, Intervertebral fusion, Osteogenesis, Bone tissue engineering

## Abstract

**Background:**

Intervertebral fusion is the most common surgery to treat lumbar degenerative disease (LDD). And the graft material used in the operation is derived from the iliac crest to promote fusion. However, autografts possess the fatal disadvantage of lack of source. Therefore, economical and practical bone substitutes are urgently needed to be developed. Sinusoidal electromagnetic fields (EMF) combined with tissue engineering techniques may be an appropriate way to promote intervertebral fusion.

**Methods:**

In this research, porous scaffolds made of polycaprolactone (PCL) and nano-hydroxyapatite (nHA) were used as cell carriers. Then, the scaffolds loaded with bone marrow mesenchymal stem cells (BMSCs) were treated with sinusoidal electromagnetic field and the osteogenic capability of BMSCs was tested later. In addition, an intervertebral disc of the tail vertebra of the rat was removed to construct a spinal intervertebral fusion model with a cell-scaffold implanted. The intervertebral fusion was observed and analyzed by X-ray, micro-CT, and histological methods.

**Results:**

BMSCs stimulated by EMF possess splendid osteogenic capability under an osteogenic medium (OM) in vitro. And the conditioned medium of BMSCs treated with EMF can further promote osteogenic differentiation of the primitive BMSCs. Mechanistically, EMF regulates BMSCs via BMP/Smad and mitogen-activated protein kinase (MAPK)-associated p38 signaling pathways. In vivo experiments revealed that the scaffold loaded with BMSCs stimulated by EMF accelerated intervertebral fusion successfully.

**Conclusion:**

In summary, EMF accelerated intervertebral fusion by improving the osteogenic capacity of BMSCs seeded on scaffolds and might boost the paracrine function of BMSCs to promote osteogenic differentiation of the homing BMSCs at the injured site. EMF combined with tissue engineering techniques may become a new clinical treatment for LDD.

## Background

According to the 2017 Global Burden of Disease Study, low back pain, headache, and depression are listed as the third-level causes of years lived with disability (YLDs) [1]. Lumbar degenerative disease (LDD) as the common etiology of low back pain is caused by the aging and degeneration of the lumbar spine. Approximately 266 million people worldwide suffer from LDD and low back pain every year [2]. With the aging of the social population, the incidence of LDD has increased year by year. Fortunately, as a recognized effective treatment, interbody fusion surgery can effectively relieve the pain or neurological symptoms associated with LDD [3]. The surgery can be operated to remove the diseased intervertebral disc via a variety of surgical approaches [4]. Then, the insertion of an interbody material and vertebral reconstruction fixation are conducted to promote intervertebral fusion. At present, autologous iliac crest bone grafts (ICBGs) are considered the gold standard due to their osteogenetic, osteoconductive, and osteoinductive potential [5]. Nevertheless, the risk of pain, bleeding, infection, and other complications in the donor site is up to 50% [6]. To make matters worse, most of the patients are the elderly who are not suitable for autologous bone transplantation due to severe osteoporosis. For multi-level intervertebral fusion surgery, the autologous bone cannot meet the amount of bone required for surgery. Therefore, the allogeneic bone is often used as a supplement in clinical practice. But its high price and possible immune response limit its wide application [7].

The urgent demand for bone substitutes contributed to the booming of the artificially synthesized materials. Now, synthetic grafts mainly include three types: (1) metal materials, such as stainless steel, titanium alloys, etc. [8]; (2) ceramic materials, such as hydroxyapatite (HA), alumina ceramics, etc. [9]; and (3) polymer materials, which are divided into natural polymers such as gelatin and synthetic biodegradable polymers such as polycaprolactone (PCL) [10].

HA is the main inorganic component of the bone with crackajack biocompatibility and the characteristics of osteoconduction and osteoinduction [11]. PCL is well known for its degradability, renewability, and good mechanical properties [12–14]. It has been widely reported that a PCL/HA composite material demonstrated enhanced osteogenic ability in bone repair [15–17]. Therefore, in our experiment, a porous scaffold made of PCL and nano-hydroxyapatite (nHA) via 3D printing technology was used as a cell carrier.

Currently, bone marrow mesenchymal stem cells (BMSCs) are the most prevalent source of stem cells in bone tissue engineering due to their relatively easy acquiring and excellent regenerative properties [18, 19]. And it has become a new trend to regulate stem cells through external intervention.

Electromagnetic fields (EMF) have been successfully employed as adjunctive therapy for the treatment of fresh fractures, osteoporosis, and spinal cord injury in the past decades [20–22]*.* Nevertheless, there is strong evidence that long-term exposure to EMF is a risk factor for many diseases and even cancer [23], which may attribute to genotoxic effects, neurological effects, and carcinogenicity of EMF [24–26]. For more secure use of EMF, its role in clinical therapy may be changed. So in this study, BMSCs seeded on scaffolds were stimulated by EMF (15 Hz, 0.3 mT) in vitro and the osteogenic indicators were examined at gene and protein levels later. The cell signaling pathways related to osteogenesis were investigated to reveal the regulatory mechanisms of EMF. To explore the effect of EMF on paracrine function of BMSCs, the conditioned medium of BMSCs treated with EMF was used for further culture of the undifferentiated BMSCs. As for in vivo experiment, a spinal fusion model was constructed by removing the intervertebral discs of rats to simulate clinical operations. Then, different tissue-engineered bones were implanted and fixed with reasonable means. The capacity of the implants to promote intervertebral fusion was evaluated and analyzed through imaging techniques and histological methods 3 months later. All the studies that had been conducted focused on accelerating intervertebral fusion via combined application of low-frequency EMF and tissue engineering techniques.

## Materials and methods

### Preparation of PCL/nHA scaffolds

PCL and nHA were mixed in dichloromethane at a ratio of 7:3. After the dichloromethane was partially volatilized, the mixture was printed into a porous scaffold with a fused deposition modeling (FDM) 3D printer. The manufactured scaffolds were divided into two types: one is a square with the side length of 8 mm, another is a disc with a diameter of 4 mm, and the thickness of all scaffolds is 1 mm. The square scaffold serves as a carrier for cells in vitro while the disc scaffold is used in vivo. Then, the scaffolds were placed in a fume hood overnight to completely volatilize the residual dichloromethane. Finally, these scaffolds were sterilized by ethylene oxide in the hospital.

### Characterization of PCL/nHA scaffolds

The porosity of PCL/nHA composite scaffolds was measured as described before [27]. Briefly, apparent volume (*V*_a_) and dry weight (*W*_d_) of the porous scaffolds (*n* = 6) were measured before they were immersed in 95% ethanol for 5 min. The scaffolds were washed with distilled water and kept in distilled water overnight to determine the wet weight (*W*_w_) of the scaffolds. *p* indicated the density of the distilled water and the scaffold porosity was calculated as: porosity (%) = $\frac{w_{\mathrm{w}}-{w}_{\mathrm{d}}}{p{v}_{\mathrm{a}.}}$

Instron 5566 device (Instron Corporation, USA) was utilized to evaluate the mechanical properties of scaffolds. Concisely, cubic specimens (*n* = 6) were placed vertically between two solid platens. The compression rate was set as 1 mm/min with a 5-N load cell. And the compression strength was acquired from the stress-strain curve.

### Cell culture

Rat bone marrow mesenchymal stem cells (BMSCs) purchased from Cyagen Biotechnology Co., Ltd. (Suzhou, China) were maintained in a α-MEM medium supplemented with 10% fetal bovine serum (Gibco, 10091148, NY, USA) and 1% of an antibiotic-antimycotic solution (Sigma-Aldrich, A5955, USA) at 37 °C, 5% CO_2_, and 95% humidity. When it was necessary to induce osteogenic differentiation of BMSCs of passage 3, we replaced the α-MEM medium with the osteogenic medium (OM) which was prepared by adding 10 nM dexamethasone, 10 mM β-glycerophosphate, and 50 mg/ml ascorbic acid to the α-MEM complete medium. The culture medium was refreshed every 3 days.

### Cell seeding

The sterilized scaffolds were placed in a 24-well plate under sterile conditions. Then, they were washed 3 times with phosphate-buffered saline (PBS; Boster, pyg0021, Wuhan, China) and bathed in the α-MEM medium without FBS for 2 h before cells seeding. Then, 6 × 10^4^ BMSCs were seeded into a 24-well plate in which a scaffold was ensconced. After the cells adhered to the scaffolds completely, the cell-seeded scaffolds were transferred to a new 24-well plate for further culture. Immediately after, these cell-scaffolds were randomly divided into two groups. The group treated with EMF was presented as the EMF group, while the group without any intervention was regarded as the control group (Ctrl group).

### Sinusoidal electromagnetic field stimulation system

**Fig. 1:**
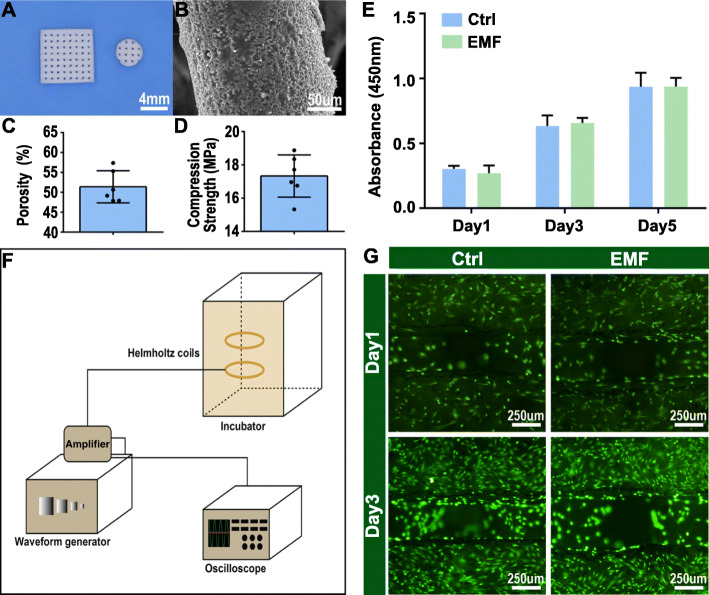
The characteristics of the scaffolds. **a** Gross observation of the PCL/HA composite scaffolds made by a 3D printer. **b** Micro-morphology and microstructure of the composite scaffolds. **c** Porosity of the scaffolds. **d** Compression strength of the scaffolds. **e** The proliferation of BMSCs after culturing for 1, 3, and 5 days on the scaffolds detected by the CCK-8 kit. **f** Presentation of the device used to generate sinusoidal electromagnetic fields (EMF). The device was constituted of the waveform generator, amplifier, oscilloscope, and Helmholtz coils. Cell-scaffolds were placed in the center of the Helmholtz coils, which were placed in a 5% CO_2_ and 37 °C incubator. **g** Live-dead staining images of BMSCs after culturing for 1 day and 3 days on the scaffolds. *p < 0.05 compared to the Ctrl group, **p < 0.01 compared to the Ctrl group

The EMF stimulation system (Fig. 1g) that has been introduced in our previous reports is made up of a EMF generator, an amplifier, an oscilloscope, and a pair of Helmhotz coil [28–30]. It was designed and manufactured by the Naval University of Engineering (Wuhan, China). Cell-seeded scaffolds from the EMF group were exposed to the sinusoidal electromagnetic field (15 Hz, 0.3 mT) for 4 h every day.

### Cell adhesion and morphology

After a week of incubation, the cells on the scaffolds were washed twice with PBS and then fixed with 2.5% glutaraldehyde at 4 °C for 4 h. Part of the cell-seeded scaffolds were stained with TRITC-Phalloidin (Solarbio, CA1610, Beijing, China) and DAPI (Solarbio, C0060, Beijing, China) after being permeabilized with 0.5% Triton X-100 solution (Beyotime, P0096, China). And the fluorescent images were captured by a confocal microscope (Eclipse, NIKON, Japan). Meanwhile, the remaining scaffolds were dehydrated through a gradient of ethanol (50, 60, 70, 80, 90, and 100%), followed by drying in a vacuum. The specimens were gold-coated and observed with scanning electron microscopy (SEM, VEGA 3 LMU, Tescan, CZ).

### Cell viability

BMSC proliferation was assessed using Cell Counting Kit-8 assay (CCK-8, Dojindo, CK04, Japan) after culturing for 1, 3, and 5 days. At each time point, 2-ml CCK-8 working solution (diluted 1:9 in fresh culture medium) was added to each well, then they were incubated at 37 °C for 2 h. The absorbance of the resulting solution was measured at 450 nm using a microplate reader (Bio-Rad, USA). The viability of BMSCs cultured on scaffolds was assessed by a Live-dead kit (Thermo Fisher, L3224, USA). On the 1st and 3rd day of culture, the scaffolds were washed twice with PBS and enough working solution was added to submerge the scaffolds. After incubation in dark for 30 min, the specimens were rinsed with PBS and observed via a fluorescence microscope (EVOS FL Auto, Life Technologies, USA). The live cells staining green while dead cells staining red.

### Alkaline phosphatase (ALP), collagen, and mineralization assay

In order to facilitate the observation of staining, 10^4^ BMSCs were seeded in 24-well plates and cultured with an osteogenic medium (OM). After a week of culture, the cells were fixed by 4% paraformaldehyde. Sirius Red (Sigma, 365548, USA) staining was used to determine the collagen deposition while ALP was stained with BCIP/NBT alkaline phosphatase Color Development Kit (Beyotime, C3206, China). In addition, 5 × 10^3^ BMSCs were seeded in 24-well plates and stained with Alizarin Red (Cyagen, China) after 2 weeks of culture. Staining positive area fraction calculated as staining positive area/total area was obtained using ImageJ software (*n* = 6).

### Osteogenesis-related gene expression of BMSCs cultured on the scaffolds

2 × 10^5^ BMSCs were seeded into a 24-well plate positioned with a scaffold, and total RNA was isolated from the BMSCs cultured for 4 days using the RNeasy Kit (Omega, R6834-01, USA). Complementary DNA (cDNA) was synthesized from 1 μg of total RNA with the help of a Reverse Transcriptase Kit (Toyobo, FSQ-101, Japan) following the manufacturer’s instructions. Expressions of genes of interest [bone morphogenetic protein 2 (BMP2), alkaline phosphatase (ALP), osteopontin (OPN), TAK1-binding protein 1 (TAB1), p38, type IB BMP receptor (BMPR1B), and Smad1/5/8] were assayed by quantitative real-time PCR (RT-qPCR). Gene-specific primers (Table 1) purchased from Tsingke Biotechnology company (Beijing, China) were used to amplify the cDNA in a Bio-Rad myiQ2 thermal cycler (Bio-Rad, Hercules, CA, USA). The 2^**–△△Ct**^ method was used to analyze the relative expression of target mRNA expression. By the way, GAPDH was used as the internal control for target mRNA.

**Table 1:**
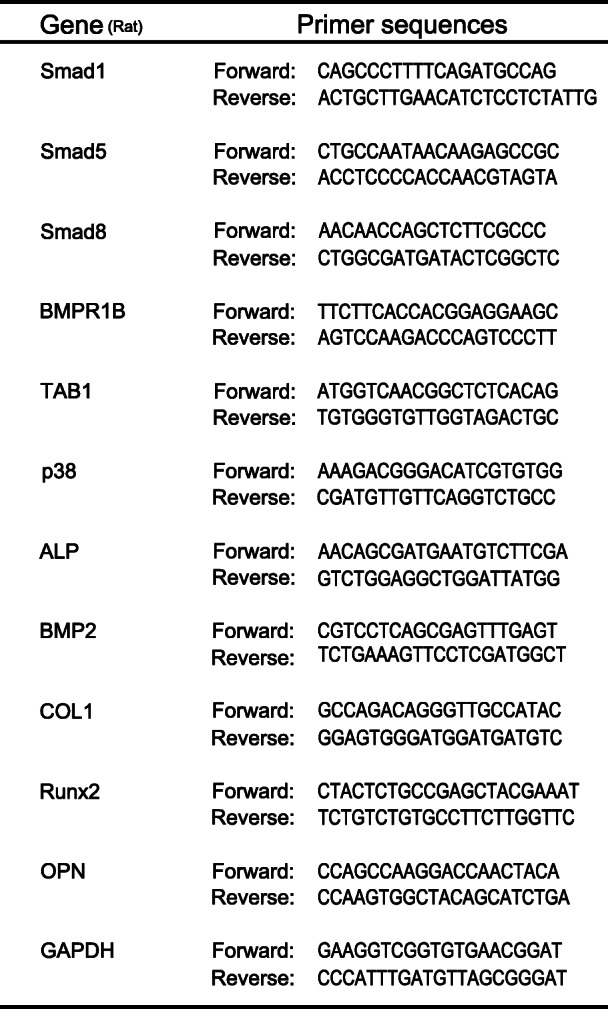
List of primer sequences used in this study

### Western blot analysis

The proteins were analyzed from whole-cell lysates of BMSCs cultured with OM after 1 week. BCA protein assay reagent (Boster, AR0197, Wuhan, China) was used to measure the protein concentration. Next, 40-μg protein samples were separated by SDS-polyacrylamide gels and transferred to PVDF membranes. The membranes were then blocked with 5% bone serum albumin (BSA) for 1.5 h and incubated with primary antibodies (COL1, BMP2 at 1:1000 dilution, β-actin at 1:5000 dilution, Abcam, UK; OCN at 1:1000 dilution, Santa Cruz Biotechnology) at 4 °C overnight. Blots were then incubated with secondary antibodies for 1 h before they were detected by the Western ECL Substrate Kit (Thermo Pierce, USA), while the proteins were normalized by β-actin.

### Immunofluorescence

BMSCs seeded on coverslips were fixed in 4% paraformaldehyde and treated with 0.5% Triton X-100 for 15 min. After being rinsed twice with PBS, the cells were blocked with 5% BSA for 30 min at room temperature. Soon afterwards, the cells were incubated with primary antibodies [rabbit anti-Runx2 (1:100, Cell Signaling Technology), mouse anti-OPN (1:50, Santa Cruz Biotechnology), rabbit anti-phospho-Smad1/5/8 (1:100, Cell Signaling Technology), mouse anti-BMPRIB (1:50, Santa Cruz Biotechnology), rabbit anti-p38 MAPK (1:50, Cell Signaling Technology), mouse anti-TAB1 (1:50, Santa Cruz Biotechnology)] against the target proteins at 4 °C overnight. Secondary antibodies including CY3-conjugated goat anti-rabbit IgG (1:200, Boster, China) and FITC-labeled goat anti-mouse IgG (1:200, Boster, China) were used to bind the primary antibodies. The fluorescence images were acquired under a confocal microscope (Eclipse, NIKON, Japan).

### BMSCs cultured with the conditioned medium

After the cell-scaffold structures were cultured for 4 days under the OM with or without EMF stimulation, the medium was discarded and the cell-seeded scaffolds were washed twice with PBS. Subsequently, the α-MEM medium was used to incubate cells seeded on scaffolds for another 4 days in a normal environment. Then, the medium was collected and 10% FBS was added to make a conditioned medium for the culture of the undifferentiated BMSCs. In addition, the undifferentiated BMSCs cultured with α-MEM served as a blank group. After 1 week of culture, the expression of osteogenic genes [Runt-related gene 2 (Runx2), type 1 collagen (COL1), and osteopontin (OPN)] were analyzed by qPCR and the osteogenic proteins (Runx2, OPN) were detected by immunofluorescence.

### Construction of the intervertebral fusion model

To prepare the cell-scaffold complex for use in vivo, 2 × 10^5^ cells were seeded into a 24-well plate positioned with a disc scaffold, and they were cultured for a week in OM with or without EMF. Then, 24 male SD rats (3 months, 300–350 g) provided from the Laboratory Animal Center of Tongji Hospital had been approved by the Animal Care and Use Committee of Huazhong University of Science and Technology. And they were used to create the intervertebral fusion model for in vivo research. First, the rats were anesthetized by intraperitoneal injection of 1% pentobarbital sodium and the hair around the tail was shaved. The skin and subcutaneous tissue were incised after disinfection to expose the vertebral bodies. The tissue around the specified vertebral body was separated and the spinous processes were excised. Next, a scalpel and ophthalmic scissors were used to completely remove the intervertebral disc with the endplates being polished by a high-speed bur. The grafts were inserted into the interbody space before the vertebral bodies were fixed with plates and screws. Finally, the incision was sutured and antibiotics were injected to prevent infection. According to the difference of implants, the rats were divided into four groups: (1) blank group: nothing was filled in the interbody space; (2) scaffold group: only scaffolds were implanted in the body; (3) S+Cell group: the cell-scaffolds cultured with OM were inserted into the interbody space; and (4) S+C+EMF group: rats were implanted with cell-scaffolds stimulated by EMF under the OM.

### Imaging tools to evaluate the intervertebral fusion

X-rays were employed to observe the intervertebral fusion 1 week, 4 weeks, 8 weeks, and 12 weeks after surgery. During the detection, isoflurane-anesthetized rats were placed in the right lateral position and the X-ray settings were as follows: voltage 42 kV, electric current 320 mA, and exposure time of 8 ms. The relative new bone mass was analyzed by Mimics software. Then, the rats were euthanized at 12 weeks postoperation and the caudal vertebrae of the operated segment were soaked in 4% paraformaldehyde. The internal-fixation plates and the surrounding soft tissues were carefully removed prior to the computed tomography (CT) scanning. Micro-CT (vivaCT 40, Scanco 274 Medical, Switzerland) scanning was performed using the following conditions: 70 mA, 120 kV, and 15 μm. All 3D images were reconstructed by Mimics (a highly integrated and easy-to-use 3D image generation and editing software). The bone volume relative to total volume (BV/TV) and the bone mineral density (BMD) within the defect area were calculated by Mimics software (*n* = 6).

### Histological methods to assess intervertebral fusion

Following all the CT scans, the specimens were immersed in 10% EDTA decalcification solution. Then, the decalcified specimens were treated with gradient alcohol dehydration and embedded in paraffin to make tissue sections. Hematoxylin and eosin (HE) staining and Masson staining were performed to analyze the new bone formation in the interbody space. New bone area fraction calculated as new bone area/defect area within the defect of each section was obtained using ImageJ software (*n* = 6).

### Statistical analysis

To determine whether the differences between the two sets of data are statistically significant, a two-tailed homoscedastic *t* test was applied. *^, #, &^*p* < 0.05 was considered to be statistically significant and **^, ##, &&^*p* < 0.01 was considered to be extremely significant; otherwise, it is not significant. Values are reported as the mean ± standard deviation (SD). All in vitro experiments were at least performed three times. In animal experiments, the power calculated by GPower software is higher than 80%, which indicated the probability of avoiding type 2 error is higher than 80%. Therefore, when the sample size is 6, the experimental conclusion is reliable.

## Results

### Characterization of PCL/HA scaffolds

The PCL/HA scaffold manufactured by a 3D printer is a porous scaffold with the side length of 8 mm and thickness of 1 mm (Fig. 1a). Cells were seeded on such a scaffold and treated with EMF in vitro. Disc-shaped scaffolds with 4-mm diameter were utilized in animal experiments (Fig. 1a). And the pore size of two types of scaffold is around 280 μm. The micro-morphology and microstructure of the composite scaffolds were observed through SEM (Fig. 1b). In addition, the porosity of PCL/HA composite scaffolds was 51.38 ± 4.03% (Fig. 1c) and stress-strain curves illustrated the porous scaffolds possessed a compression strength of 17.33 ± 1.27 MPa (Fig. 1d).

### Effects of EMF on cell viability and morphology

**Fig. 2:**
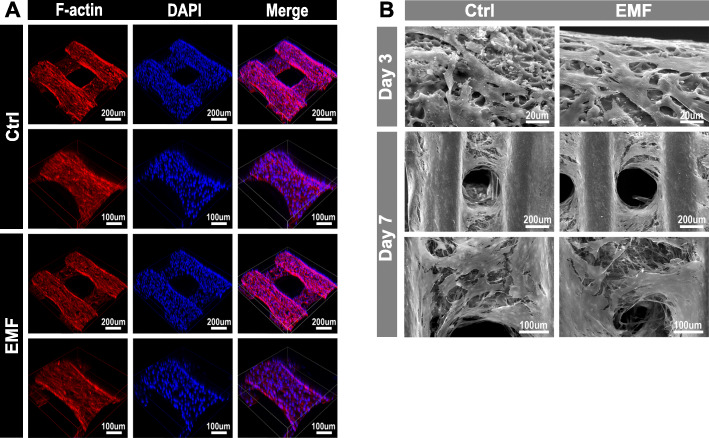
Distribution and morphology of BMSCs. **a** Distribution of BMSCs seeded on the scaffold treated with or without EMF for 1 week observed by a confocal microscope. **b** Morphology of BMSCs seeded on the scaffold after culturing for 3 and 7 days observed by SEM

Cells were cultured for 1, 3, and 5 days for cellular proliferation assay. The results (Fig. 1e) demonstrated the cells proliferated over time and there was no significant difference between the two groups at each time point. Simultaneously, the live/dead staining revealed EMF did not affect the activity of BMSCs (Fig. 1g). Confocal microscope (Fig. 2a) and SEM (Fig. 2b) were used to observe the morphology and distribution of the cells at 1 week from cell seeding. As shown in Fig. 2b, cells were spread and intercellular connections were maintained through cytoplasmic elongations. A large amount of connected cells densely cover the surface of the scaffold. Moreover, cells were evenly distributed on the scaffold regardless of EMF stimulation.

### Effects of EMF on osteogenic differentiation of BMSCs

**Fig. 3:**
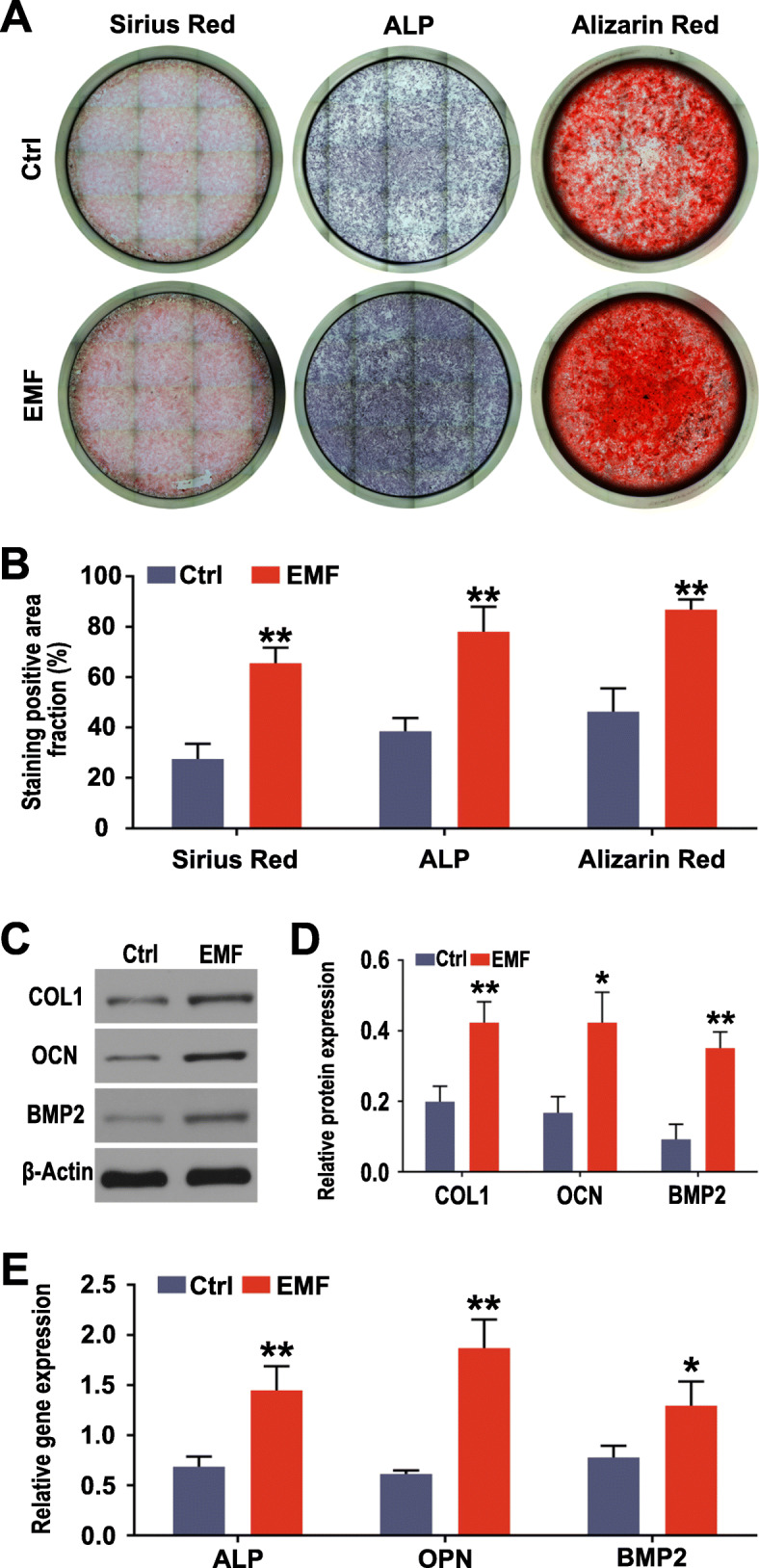
Regulation of EMF on osteogenic differentiation of BMSCs. **a** Images of ALP and Sirius Red staining of BMSCs after culturing for 1 week; images of Alizarin Red staining of BMSCs after culturing for 2 weeks. **b** Semi-quantitative analysis of ALP, Sirius Red, and Alizarin Red staining among both groups (*n* = 6). Data are shown as mean ± SD. **c** Effects of EMF on osteogenic protein expression of BMSCs after culturing for 1 week (COL1, OCN, and BMP2) detected by western blotting. **d** Quantification analysis of the osteogenic protein expression (COL1, OCN, and BMP2) (*n* = 3). Data are shown as mean ± SD. **e** Effects of EMF on osteogenic gene expression of BMSCs after culturing for 4 days (ALP, OPN, and BMP2) detected by RT-qPCR (*n* = 3). *p < 0.05 compared to the Ctrl group, **p < 0.01 compared to the Ctrl group

**Fig. 4:**
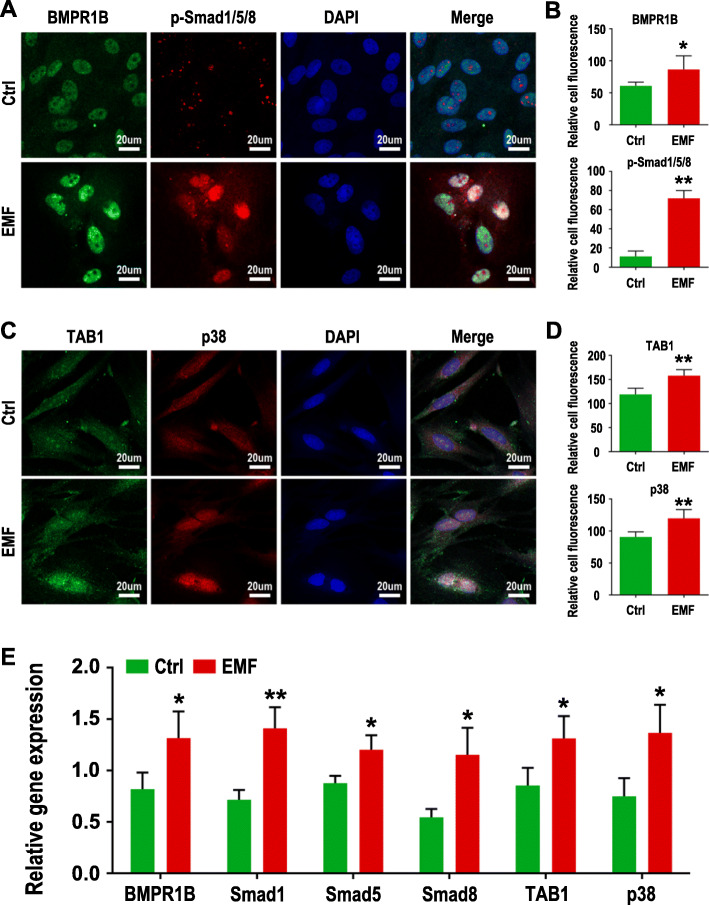
Regulation of EMF on BMP/Smad and TAB1/p38 MAPK signaling pathways. **a** Immunofluorescence labeling for BMPR1B and P-Smad1/5/8 of BMSCs cultured for 4 days. **b** Fluorescence quantitative analysis of BMPR1B and P-Smad1/5/8 (*n* = 5). **c** Immunofluorescence labeling for TAB1 and p38 of BMSCs cultured for 4 days. **d** Fluorescence quantitative analysis of TAB1 and p38 (*n* = 5). **e** Gene expression of BMPR1B, Smad1/5/8, TAB1, and p38 of BMSCs was detected by RT-qPCR (*n* = 3). *p < 0.05 compared to the Ctrl group, **p < 0.01 compared to the Ctrl group

Osteogenesis is the core of intervertebral fusion. A series of experiments were performed to explore osteogenic differentiation of BMSCs treated with EMF. ALP staining kit and Direct Red 80 were employed to test ALP and collagen deposition, respectively. As shown in Fig. 3a and b, the cells from the EMF group were stained with a darker color, which means a higher expression of osteogenic indicators. In addition, the same results were obtained with Alizarin Red staining of cells cultured for 2 weeks (Fig. 3a, b). Consistent with the results of the above experiments, the relative expression level of osteogenic genes [alkaline phosphatase (ALP), bone morphogenetic protein 2 (BMP2), and osteopontin (OPN)] (Fig. 3e) and osteogenic proteins [type I collagen (COL1), BMP2, and osteocalcin (OCN)] (Fig. 3c, d) also confirmed that BMSCs treated with EMF showed more significant osteogenic differentiation under OM. Moreover, the results of immunofluorescence (Fig. 4a–d) and RT-qPCR (Fig. 4e) both suggested that EMF regulate differentiation of BMSCs by activating BMP/Smad and TAB1/p38 MAPK signaling pathways.

### Effects of conditioned medium of BMSCs treated with EMF

**Fig. 5:**
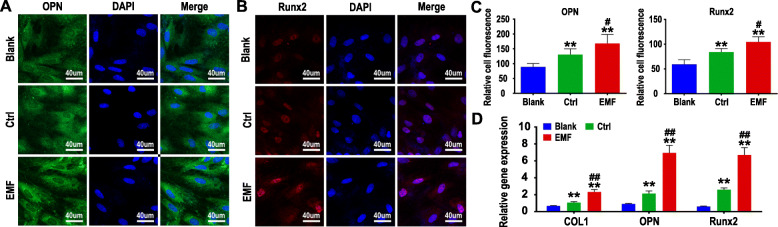
Effects of the conditioned medium of BMSCs on undifferentiated BMSCs. **a** Immunofluorescence labeling for OPN of BMSCs cultured with the conditioned medium for 1 week. **b** Immunofluorescence labeling for Runx2 of BMSCs cultured with the conditioned medium for 1 week. **c** Fluorescence quantitative analysis of OPN and Runx2 (*n* = 5). **d** Gene expression of COL1, OPN, and Runx2 of BMSCs cultured with the conditioned medium was detected by RT-qPCR (*n* = 3). *p < 0.05 compared to the Blank group, **p < 0.01 compared to the Blank group, ^#^*p* < 0.05 compared to the Ctrl group, ^##^*p* < 0.01 compared to the Ctrl group

The cell-scaffold structures will face a complex environment after being implanted. To investigate the effect of EMF-stimulated BMSCs on undifferentiated BMSCs in surrounding tissues, the conditioned medium of BMSCs treated with EMF was collected to cultivate a new batch of BMSCs. As indicated in Fig. 5d, the conditioned mediums significantly upregulated the expression of osteogenic genes of BMSCs compared with the blank group. Moreover, the conditioned medium of EMF-stimulated BMSCs possessed a better effect of promoting osteogenic differentiation than the conditioned medium of BMSCs, which may attribute to the enhancement of the paracrine function of BMSCs by EMF, resulting in the release of more osteogenic cytokines into the medium. The results of immunofluorescence on OPN and Runx2 (Fig. 5a–c) also support this argument.

### Construction of intervertebral fusion model and X-ray assessment

**Fig. 6:**
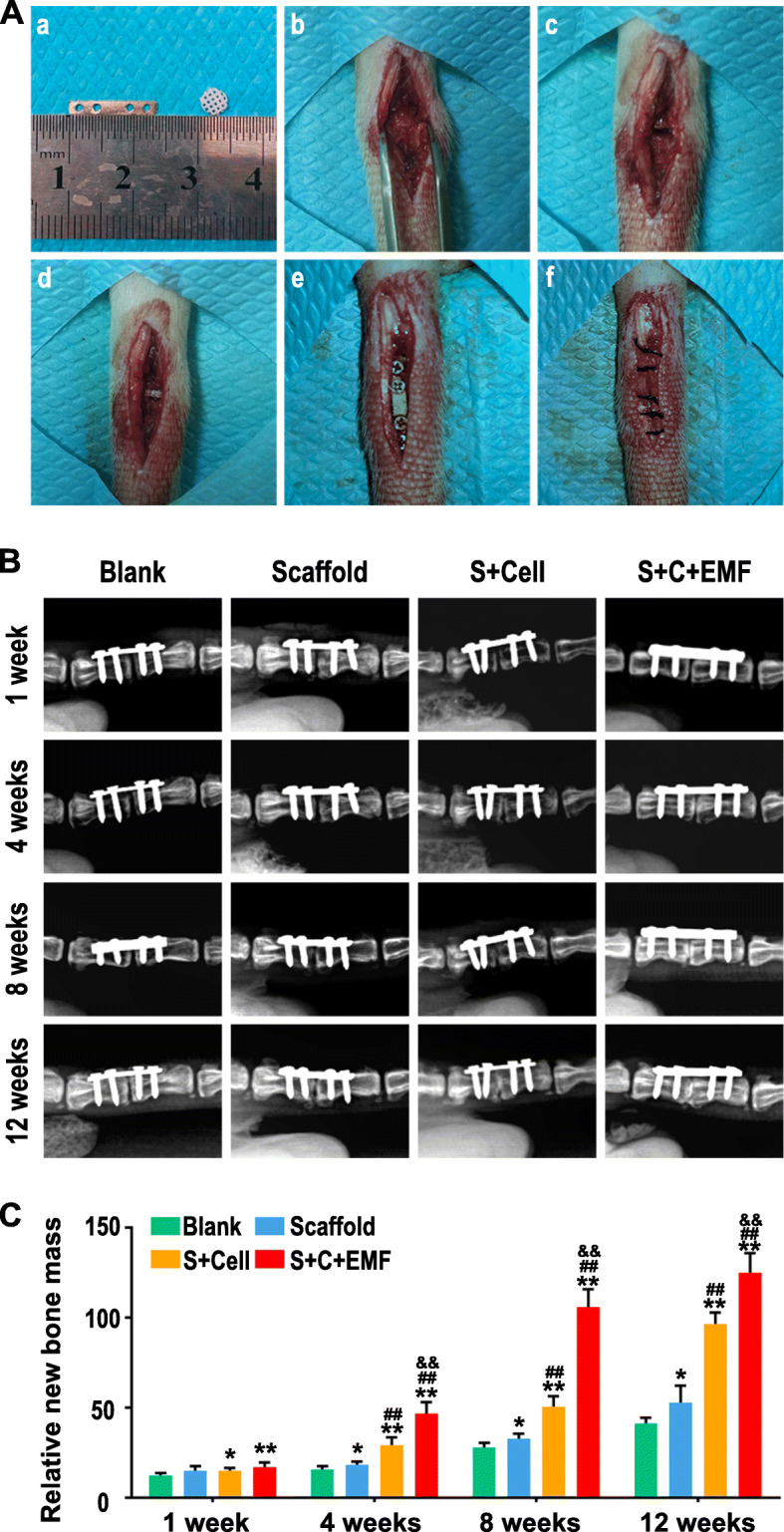
In vivo experiments. **A** The key steps to establish the intervertebral fusion model of rats. (a) The preparation of internal-fixation plates and scaffolds. (b) The skin and subcutaneous tissue were incised to expose the vertebral bodies. (c) The intervertebral disc was completely removed. (d) The cell-scaffold was inserted into the interbody space. (e) The vertebral bodies were fixed with plates and screws. (f) The incision was stitched with sutures. **B** Representative X-ray evaluation of intervertebral fusion in different groups taken 1, 4, 8, and 12 weeks after surgery. **C** The quantitative analysis of X-ray analyzed by Mimics software (*n* = 6). *p < 0.05 compared to the Blank group, **p < 0.01 compared to the Blank group, ^#^*p* < 0.05 compared to the Scaffold group, ^##^*p* < 0.01 compared to the Scaffold group, ^&^*p* < 0.05 compared to the S+Cell group, ^&&^*p* < 0.01 compared to the S+Cell group

The key steps to establish the animal model are shown in Fig. 6A. For X-ray assessment in Fig. 6B and C, the vertebral bodies adjacent to the defect site did not demonstrate a fusion tendency in the blank group. The defects in the Scaffold group and S+Cell group were almost repaired 12 weeks after the operation, leaving only a narrow gap between adjacent vertebrae. Moreover, the intervertebral fusion of S+Cell group was slightly better than that of the Scaffold group. However, the S+C+EMF group showed a significant intervertebral fusion tendency 8 weeks after the operation and the adjacent vertebral bodies were completely fused to each other at 12 weeks postoperatively.

### Micro-CT evaluation

**Fig. 7:**
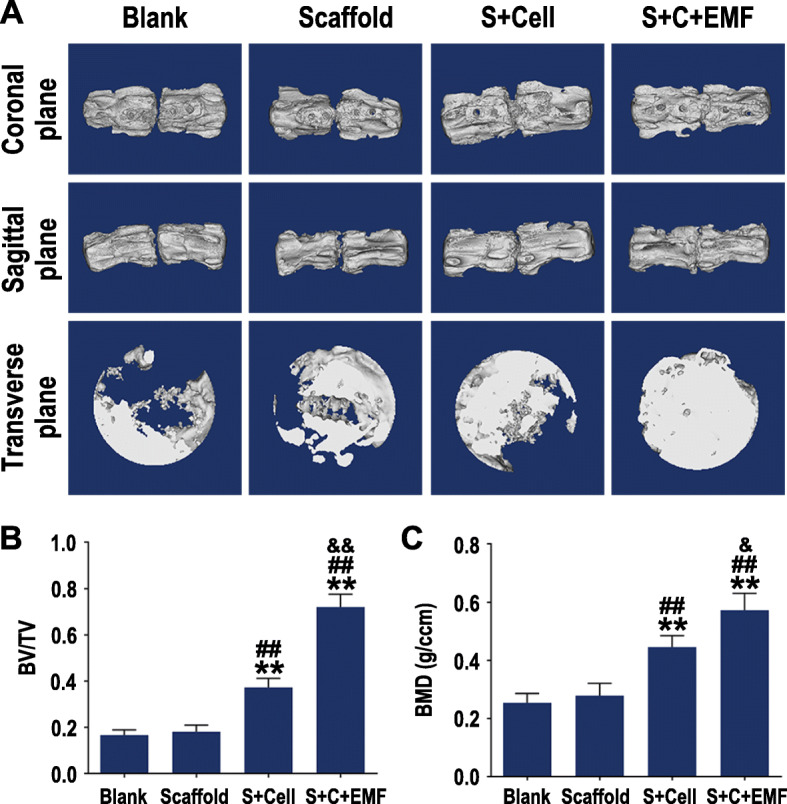
Bone regeneration evaluated by micro-CT. **a** 3D reconstructed micro-CT images of the vertebral bodies in different groups at 12 weeks. **b** BV/TV and **c** BMD quantification analysis in each group (*n* = 6). *p < 0.05 compared to the Blank group, **p < 0.01 compared to the Blank group, ^#^*p* < 0.05 compared to the Scaffold group, ^##^*p* < 0.01 compared to the Scaffold group, ^&^*p* < 0.05 compared to the S+Cell group, ^&&^*p* < 0.01 compared to S+Cell group

The reconstructed 3D images (Fig. 7a) and statistical data (Fig. 7b, c) stated the results consistent with the X-ray assessment. In brief, limited bone formation was observed in the blank group at 12 weeks. For the Scaffold group, a small amount of new bone formation is not sufficient to facilitate intervertebral fusion. As shown in S+Cell and S+C+EMF groups, the adjacent vertebral bodies had fused with each other, but the S+C+EMF group possessed a more complete fusion region from the transverse plane.

### Histological evaluation

**Fig. 8:**
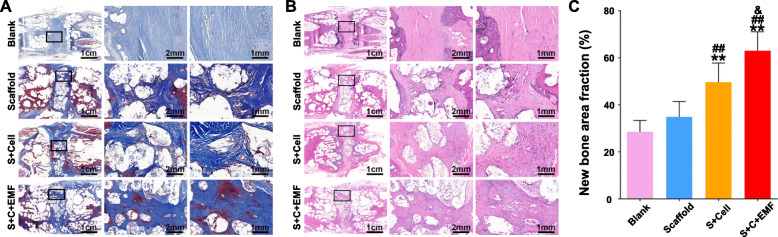
Bone regeneration evaluated by Masson’s trichrome and HE staining. **a** Masson’s trichrome staining was conducted to show the newly formed bone within the implanted constructs at 12 weeks postoperatively. **b** HE staining was conducted to show the newly formed bone within the implanted constructs at 12 weeks postoperatively. **c** Quantification analysis of new bone area fraction in different groups at 12 weeks (*n* = 6). *p < 0.05 compared to the Blank group, **p < 0.01 compared to the Blank group, ^#^*p* < 0.05 compared to the Scaffold group, ^##^*p* < 0.01 compared to the Scaffold group, ^&^*p* < 0.05 compared to the S+Cell group, ^&&^*p* < 0.01 compared to the S+Cell group

New bone formation in the interbody space was assessed with Masson’s trichrome staining 12 weeks after implantation (Fig. 8a, c). For the blank group, a lot of fibrous connective tissue filled the interbody space with little new bone formation. A small amount of newly formed bone was observed in the Scaffold group, while the S+Cell group exhibited a better bone regeneration compared to the above groups. In the S+C+EMF group, there was a large amount of newly formed bone in the interbody space. Furthermore, partial vertebral fusion has been observed at the periphery of the interbody space, leaving some undegraded scaffolds in the center. HE staining (Fig. 8b, c) was also employed to evaluate the intervertebral fusion and consistent tendency was observed. All evidence suggested that EMF combined with tissue engineering techniques can accelerate intervertebral fusion.

## Discussion

Lumbar degenerative disease as a common ailment which causes pain and disability in patients has troubled human beings for many years. The benefits of intervertebral fusion surgery have been demonstrated in the large number of patients as evidenced by lowered pain and disability scores and the ability to return to work [31]. Although the ICBGs have been considered the gold standard as a source of graft for lumbar fusion surgery, the use of ICBGs is associated with morbidity and complications [32]. So the optimal alternative to autograft bone is still under debate. In the past decades, a significant number of novel materials have been exploited with the development of science and technology. Interbody cages designed with polyetheretherketone (PEEK) have been commonly endorsed with excellent clinical outcomes in recent years [33]. However, the nondegradable property of the PEEK may lead to the risk of long-term complications and surgical intervention for implant removal [34]. In vitro studies demonstrate titanium creates an environment supportive of osteoblastic activity compared to PEEK and nanometric roughening of the titanium surface can encourage bone ingrowth and adhesion [35], while the fusion advantages and theoretical bone growth of titanium have not been adequately identified [36].

Besides the materials, stem cells, growth factors, and mechanical stimulation also play an indispensable role in bone tissue engineering [37]. A variety of growth factors such as BMP, VEGF, and FGF have been widely explored for application in recent years. Nevertheless, the outcomes are far from satisfaction due to the high cost and the fast clearance in vivo [38]. The main goal of the addition of growth factors is to promote osteogenesis and angiogenesis. Our previous studies have shown that low-frequency sinusoidal electromagnetic fields have the ability to promote osteogenesis and angiogenesis of BMSCs [30]. So the sinusoidal electromagnetic fields as a safe and noninvasive physical intervention is likely to be a substitute for growth factors in the future. This study verified the regulation of EMF on BMSCs in osteogenesis once again and was the first to utilize scaffolds loaded with EMF-stimulated BMSCs to promote intervertebral fusion.

The results of CCK-8 and live/dead assay illustrated that EMF had little consequence on proliferation and activity of BMSCs in vitro, which is consistent with Dr. Celik’s findings [39]. And EMF did not affect the morphology and distribution of BMSCs on scaffolds according to the images observed by SEM and confocal microscope. However, a series of tests on osteogenic indicators suggested that BMSCs treated with EMF demonstrated better osteogenic capacity under the osteogenic medium. Mechanistically, several signaling pathways such as BMP/Smad and MAPK-associated p38 pathways involved in osteogenic differentiation of BMSCs have been reported [40–42]. And this research illustrated that EMF could activate BMP/Smad and TAB1/p38 MAPK pathways, resulting in osteogenic differentiation of BMSCs. In addition, previous studies stated that EMF could promote the differentiation of BMSCs through the Wnt/β-catenin pathway [43]. So it can be established that the regulation of EMF on BMSCs is a multi-pathway and complex process. Furthermore, the conditioned medium of BMSCs can promote the osteogenic differentiation of the initial BMSCs. This may be attributed to the paracrine function of BMSCs [44–46]. What makes sense is that EMF further enhance this capability. When the body suffers trauma, BMSCs might move from their niche into the peripheral circulation and arrive at target tissues in response to injury signals [47, 48]. Therefore, BMSCs treated with EMF can better promote osteogenic differentiation of the homing BMSCs through paracrine in vivo, which benefits the acceleration of intervertebral fusion.

To evaluate the potential application of EMF combined with tissue engineering techniques, a spinal fusion model was created. Both radiological evidence and tissue sections demonstrated the important role of EMF-stimulated BMSCs in promoting intervertebral fusion. And it provided a strong support for the clinical practice of this therapeutic regimen. Currently, EMF as a physiotherapy plays a role of adjuvant therapy in clinical practice and patients are at risk for direct exposure to EMF [23, 49]. According to our strategy, a large-scale culture of BMSCs treated with EMF in vitro compensated for inadequate supply from the autologous bone while avoiding direct patient exposure to EMF. The combination between EMF-stimulated stem cells and tissue engineering techniques will bring gospel to patients with LDD. We believe that increasingly deeper knowledge of the EMF will permit its wider range of applications in regenerative medicine.

## Conclusions

Low-frequency sinusoidal electromagnetic fields (15 Hz, 0.3 mT) have little consequence on proliferation and activity of BMSCs, but can enhance the osteogenic capacity of BMSCs in the osteogenic microenvironment. The regulation of EMF on BMSC osteogenic differentiation is a multi-pathway and intricate process. BMP/Smad and TAB1/p38 MAPK signaling pathways have been found to be involved in the regulation of EMF on BMSCs. Furthermore, EMF can boost the paracrine function of BMSCs, so as to promote the osteogenic differentiation of homing BMSCs in vivo. EMF combined with tissue engineering techniques demonstrated excellent performance in promoting intervertebral fusion. Our research aims at providing new strategies for the clinical treatment of lumbar degenerative disease.

## Data Availability

The datasets used and/or analyzed during the current study are available from the corresponding author on reasonable request.

## Abbreviations

EMF: Electromagnetic fields
PCL: Polycaprolactone
HA: Hydroxyapatite
BMSCs: Bone marrow mesenchymal stem cells
OM: Osteogenic medium
MAPK: Mitogen-activated protein kinase
YLDs: Years lived with disability
LDD: Lumbar degenerative disease
ICBGs: Iliac crest bone grafts
FDM: Fused deposition modeling
FBS: Fetal bovine serum
PBS: Phosphate-buffered saline
SEM: Scanning electron microscopy
ALP: Alkaline phosphatase
BMP2: Bone morphogenetic protein 2
OPN: Osteopontin
BMPR1B: Type IB BMP receptor
BSA: Bone serum albumin
Runx2: Runt-related gene 2
COL1: Type 1 collagen
CT: Computed tomography
HE: Hematoxylin and eosin
BV: Bone volume
TV: Total volume
BMD: Bone mineral density
